# Chinese Herbal Medicines and Active Metabolites: Potential Antioxidant Treatments for Atherosclerosis

**DOI:** 10.3389/fphar.2021.675999

**Published:** 2021-05-13

**Authors:** Luxia Song, Jie Zhang, Runmin Lai, Qiuyi Li, Jianqing Ju, Hao Xu

**Affiliations:** ^1^ Graduate School, Beijing University of Chinese Medicine, Beijing, China; ^2^ National Clinical Research Center for Chinese Medicine Cardiology, Xiyuan Hospital, China Academy of Chinese Medical Sciences, Beijing, China

**Keywords:** oxidative stress, atherosclerosis, anti-oxidant treatment, traditional Chinese medicine, Chinese herbal medicines

## Abstract

Atherosclerosis is a complex chronic disease that occurs in the arterial wall. Oxidative stress plays a crucial role in the occurrence and progression of atherosclerotic plaques. The dominance of oxidative stress over antioxidative capacity generates excess reactive oxygen species, leading to dysfunctions of the endothelium and accelerating atherosclerotic plaque progression. Studies showed that Chinese herbal medicines and traditional Chinese medicine (TCM) might regulate oxidative stress; they have already been used to treat diseases related to atherosclerosis, including stroke and myocardial infarction. This review will summarize the mechanisms of oxidative stress in atherosclerosis and discuss studies of Chinese herbal medicines and TCM preparations treating atherosclerosis, aiming to increase understanding of TCM and stimulate research for new drugs to treat diseases associated with oxidative stress.

## Introduction

Atherosclerosis is one of the primary causes of death and is becoming one of the greatest threats to human health. Lancet Global Health reported that the number of patients with carotid plaques and stenosis worldwide increased significantly from 2000 to 2020 (Song et al., 2020). Atherosclerotic plaques cause vessel stenosis, which hinders the normal blood flow and leads to ischemia changes in tissues and organs (Willeit et al., 2003). Depending on the location of the atherosclerotic plaque, it may cause coronary artery disease, cerebrovascular disease (stroke), or peripheral arterial disease. Several factors induce atherosclerosis (Libby et al., 2019), including hypertension, hyperlipidemia, diabetes, long-term smoking, obesity; there are also non-disease factors such as gender and age (Gress et al., 2000; McClelland et al., 2006; Tyrrell and Goldstein, 2021). The pathogenesis of atherosclerosis is hypothesized to include inflammation, lipid infiltration, oxidative stress, platelet hyperfunction, immune dysfunction, and shear stress (Mury et al., 2018). Oxidative stress and inflammation are two primary factors in the progression of atherosclerosis (Hulsmans and Holvoet, 2010). When the antioxidant activity is insufficient to reduce reactive oxygen species (ROS), excess of the latter jeopardizes arterial endothelial function and threatens plaque stability (Davignon and Ganz, 2004; Laufs et al., 2005).

In this review, we collected the relevant clinical and experimental studies and reviews by searching papers published from January 2000 to February 2021 in Pubmed, Web of science, the China National Knowledge Internet (CNKI), and the China academic database, Wanfang, using “atherosclerosis”, “oxidative stress”, “TCM” or “TCM formula” or “TCM preparation” or “Chinese herbal medicine” or “herbal active compounds” or “herbal active ingredients” or “herbal monomer” as the term. We will first review the mechanism of atherosclerotic plaque formation and progression. Then we discuss oxidative stress in the development of atherosclerotic plaques. Finally, we summarize experimental and clinical research on Chinese herbal medicines, active metabolites, and TCM prescriptions to treat atherosclerosis. The purpose of our review is to summarize the efficacy and mechanism of the Chinese herbal medicines in treating atherosclerosis from the perspective of antioxidants and provide evidence and deeper insights for future drug exploration and application in this area.

## Formation and Progression of Atherosclerosis

The structure of typical arterial walls includes vascular intima, media, and adventitia. Typical atherosclerotic are characterized by intimal thickening, excessive deposition of lipid, and infiltration of monocytes and lymphocytes. Endothelial injury dysfunction initiates atherosclerosis. Endothelial cells attach to the inner sides of arterial walls, where they help mediate immune functions by expressing adhesion molecules during inflammation that mediate the removal of swallowing foreign bodies (Libby et al., 2011). NO, prostacyclin, and bradykinin are generated by endothelial cells (Davignon and Ganz, 2004); these factors dilate blood vessels and prevent white blood cell adhesion and platelet aggregation. Endothelial cells also produce endothelin and angiotensin II that regulate vasoconstriction, promoting the proliferation of smooth muscle cells and affecting plaque progression.

Hypertension (Li et al., 2020a), hyperlipidemia (Drechsler et al., 2010), chronic smoking (Naya et al., 2011), and changes in shear stress (Chatzizisis et al., 2007) are risk factors for atherosclerosis; all result in inflammation and endothelial cell dysfunction, causing changes in permeability and expression of adhesion molecules such as vascular cell adhesion molecule-1 (VCAM-1) and E-selectin (Tabas et al., 2015). Adhesion molecules recruit inflammatory monocytes to adhere to endothelial cells and infiltrate the arterial intima (Tabas et al., 2007). Low-density lipoprotein (LDL) is transported to the arterial wall by recognizing guanine nucleotide exchange factor 4 (DOCK4) and scavenger receptor type B (SR-B1) in endothelial cells and modified to oxidized LDL (ox-LDL) (Huang et al., 2019). Leukocytes transform into macrophages and express SRs such as SR-A1, lipoxygenase 1 (Lox1), and CD36, recognizing the oxidized epitope of ox-LDL and internalizing ox-LDL to form foam cells (Canton et al., 2013; Chistiakov et al., 2017). Macrophages are the primary inflammatory cells in atherosclerotic plaques and are significant in plaque formation; they modulate plaque stability by polarizing into M1 and M2 macrophages (Canton et al., 2013).

M1 macrophages secrete pro-inflammatory factors TNF-α, IL-6, IL-1β, inducible nitric oxide synthesis (iNOS), and other effectors that promote early plaque formation, thinning fibrous caps, and enhancing immune response (Moore and Tabas, 2011; Barrett et al., 2019; Liao et al., 2020). Reverse cholesterol transport (RCT) is an important mechanism that maintains balanced lipid metabolism. High-density lipoprotein (HDL) transports cholesterol from extrahepatic tissue such as foam cells and atherosclerotic plaques to the liver for catabolism (Yu et al., 2019). HDL participates in RCT and exerts antioxidant and anti-inflammatory functions. In atherosclerosis, the structure and composition of HDL changes, hindering RCT and accelerating plaque progression (Ouimet et al., 2019). LXRα (liver X receptorα)/ABCA1 (ATP binding cassette subfamily A member 1) is a critical signal in RCT. Changes in this pathway promote the entry and retention of cholesterol-containing LDL particles in the arterial wall, causing early atherosclerosis lesions characterized by accumulation of macrophages, foam cells, and lipid droplets.

**FIGURE 1:**
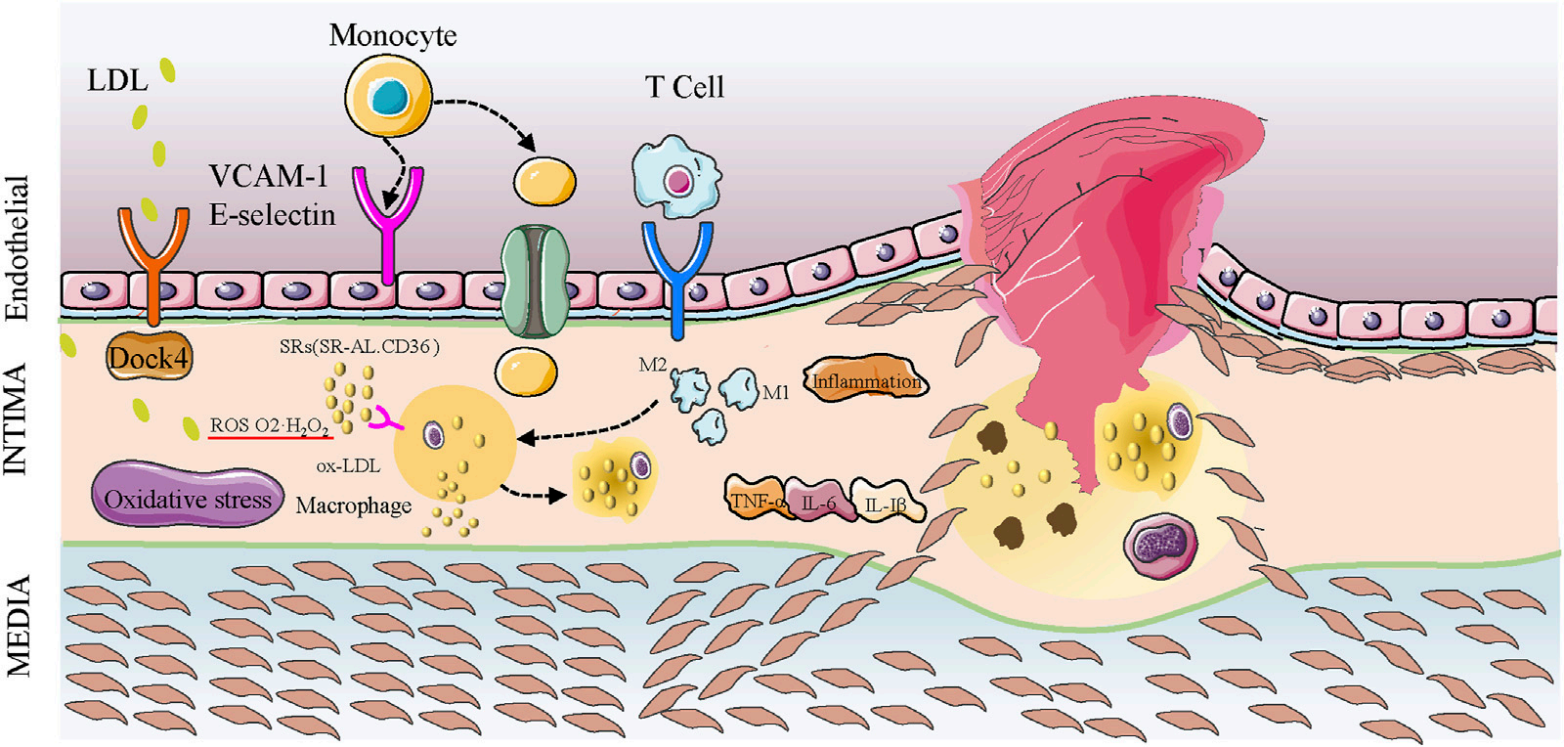
Development of atherosclerotic plaques. LDL enters the arterial intima via endothelial cells expressing SR-B1 receptors in combination with DOCK4 action. LDL particles are oxidized to ox-LDL, and the monocytes entering the intima are transformed into macrophages that phagocytize ox-LDL mediated by surface SR (SR-A1, CD36). They also phagocytize other cholesterols in the intima to form foam cells. Macrophages are polarized into M1 and M2 forms. M1 macrophages release pro-inflammatory factors such as IL-6 to promote plaque progression, and oxidative stress promotes inflammatory factors. SMCs enter the intima to form fibrous caps, and oxidative stress and inflammation promote apoptosis and cell death in the plaque, leading to the accumulation of lipid and lipid cores. The continuous inflammation and oxidative stress causes the lipid nuclei to enlarge, the fibrous cap dilutes and ruptures, and platelets accumulate to form thrombi.

In atherogenesis, smooth muscle cells (SMCs) migrate to the inner membranes of the arterial walls and secrete extracellular matrix (ECM) components such as collagen and proteoglycan. Proteoglycan regulates ECM remodeling and cytokine function, interacting with apolipoprotein B, and retaining LDL under the endothelium (Stephens et al., 2011), forming a fibrous cap with proliferated SMCs covering the plaques. The fibrous cap covers macrophage derived foam cells. As the disease continues, these foam cells undergo apoptosis, causing the accumulation of extracellular lipids to form lipid-rich plaque cores, enlarged lipid or necrotic cores, that protrude into the artery. In advanced stages of atherosclerosis, the apoptosis of SMCs and the decomposition of collagen and elastin (exacerbated by the inflammatory process (Karunakaran et al., 2021)) cause rupture of the fibrous cap around the lipid core and incite coagulation factors to interact with tissue factors, leading to thrombus formation and associated complications (Figure 1).

## Oxidative Stress and Atherosclerosis

**FIGURE 2:**
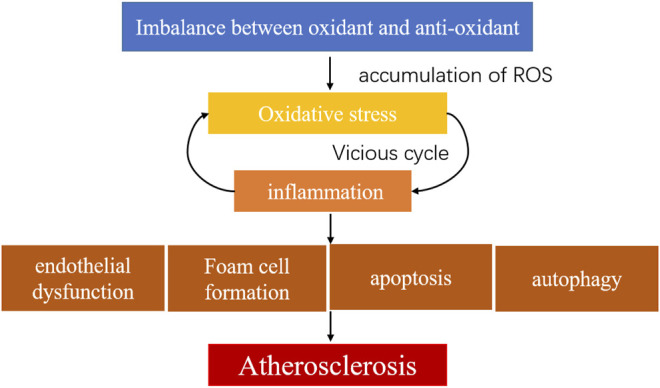
Oxidative stress in atherosclerosis.

Oxidative stress refers to the increased production of ROS in tissues or cells that weaken scavenging ability. Oxidative stress and inflammation are two major mechanisms of atherosclerosis. They interact with one another and form a vicious cycle in plaque progression. ROS include superoxide (compounds containing superoxide ion), hydrogen peroxide (H_2_O_2_), hydroxyl radicals, and nitric oxide radicals. These ROS participate in cell growth, proliferation, apoptosis, endothelial activation, mitochondrial damage, adhesion, and vascular inflammation process in atherosclerosis (Li and Shah, 2004; Madamanchi et al., 2005; Zhang et al., 2020a) (Figure 2). Major sources of ROS include nicotinamide adenine dinucleotide phosphate oxidase (NADPH oxidase or Nox), mitochondrial enzymes, Lox, uncoupled endothelial nitric oxide synthase (eNOS or NOS3), myeloperoxidases (MPO), cyclooxygenase (COX), mitochondria, and xanthine oxidase (XO). NADPH oxidase is the primary enzyme of ROS generation and has seven isoforms, namely Nox1-Nox5, Duox1, and Duox2, found in endothelial cells, vascular SMCs, fibroblasts, or perivascular adipocytes (Konior et al., 2014). Malondialdehyde (MDA), a lipid oxidation product, modifies LDL particles, leading to vascular endothelial cell structure changes and jeopardizing endothelial function. Superoxide dismutase (SOD), glutathione (GSH), catalase (CAT), paraoxonase (PON), and nitric oxide (NO) are antioxidants that degrade excessive ROS to maintain internal homeostasis. The dominance of oxidation over antioxidant capabilities leads to excess accumulation of oxygen free radicals and metabolites (Cadenas, 2018). This disequilibrium leads to oxidative stress in atherosclerotic diseases (Griendling and FitzGerald, 2003a; Griendling and FitzGerald, 2003b; Sies, 2015). ROS produced by macrophages and SMCs may participate in inflammation, endothelial dysfunction, apoptosis, autophagy, and increased plaque vulnerability (Channon, 2002; Lin et al., 2012). Over-activation of Nox increases superoxide formation and ROS generation, impairing the NO production in the arterial wall and causing vascular endothelial dysfunction. For example, eNOS is associated with arterial endothelial function. Tetrahydrobiopterin (BH4) and L-arginine are cofactors for eNOS production. Insufficient synthesis of either of these two factors cause eNOS reduction or uncoupling, impairs NO production, and accelerates superoxide accumulation, causing endothelial dysfunction and hastening atherosclerosis (Antoniades et al., 2006; Antoniades et al., 2007; Daiber and Chlopicki, 2020). However, the evidence also suggests that the endothelial isoform Nox4 produces protective H_2_O_2_, maintains endothelial function, reduces macrophage adhesion to endothelial surfaces, and provide anti-atherosclerotic functions (Schürmann et al., 2015; Langbein et al., 2016). XO produces ROS through molecular oxygen as an electron acceptor (Förstermann et al., 2017). After ROS accumulation in the arterial walls, the inhibition of xanthine dehydrogenase (XDH) and the activation of XO activity causes active oxygen production, creating a vicious cycle (McNally et al., 2003). XO also stimulates macrophages and vascular smooth muscle cells (VSMCs) to generate Lox-1 and increase ROS accumulation. Lox-1, a specific receptor for ox-LDL, activates ROS generation and NF-κB (Murdocca et al., 2021), impairing eNOS expression and causing endothelial dysfunction. ROS converts XDH into XO, causing mitochondrial damage (Zhang et al., 2020b). MPO is an oxidant highly expressed in neutrophils that produces hypochlorous acid (HOCI) from H_2_O_2_ and becomes a significant ROS generator in inflammatory response. In atherosclerosis-related diseases, MPO oxidizes apolipoprotein A1 (ApoA1) in high-density lipoprotein (HDL) (Huang et al., 2013) and impairs cholesterol acceptor function (Huang et al., 2014). Overproduction of HOCI, which MPO generates, can directly jeopardize macrophages, causing cell death and increasing plaque inflammation by recruiting neutrophils and accelerating plaque progression (Hickey, 2011; Duivenvoorden et al., 2013; Guo et al., 2020). The adhesion molecules expressed on endothelium recruit monocytes, and inflammatory cells stimulate monocytes to infiltrate into the intima, which mutually affects endothelial function. Oxidative stress occurring in plaques activates inflammatory pathways, such as NF-κB, and enhances adhesion molecule production, promoting plaque progression and thrombus formation via platelet activation (Liu et al., 2021). It also participates in macrophage polarization and increases M1 production, activating inflammation and reducing stability of vulnerable plaques (Yang et al., 2020a). ROS induces SRs in SMCs, leading to transformation into foam cells and promoting the release of matrix metalloproteinases, causing dilution of fibrous caps and plaque disruption (Kattoor et al., 2017).

Many studies examined ROS-stimulating arterial walls producing cytokines to activate signal pathways. The mechanism and critical targets of oxidative stress in the occurrence and development of atherosclerosis have also been further explored. Nrf2 eliminates ROS production, increasing the expression of antioxidant enzyme genes and maintaining oxidation/antioxidant balance in atherosclerosis (Zhu et al., 2019a). Sirtuin (Sirt) family proteins, the silent information regulators of deacetylase activity, are also crucial in the process of oxidative stress in atherosclerosis. Overexpression of Sirt1 up-regulates antioxidant-related expression, promotes the generation of transcription factor forkhead protein O subfamily 3a (FOXO3a)/PGC-1α complex, and up-regulates SOD secretion, exerting an antioxidant role against the progression of atherosclerosis (Olmos et al., 2013). Uncoupling proteins (UCPs), members of the mitochondrial transporter proteins family, act as proton transporters on the inner mitochondrial membrane related to ROS production, endothelial dysfunction, cell apoptosis, and proliferation; these also become target genes in atherosclerosis (Kim et al., 2007). An in-depth study of the specific mechanisms of oxidative stress in atherosclerosis would help identify new medications to treat atherosclerosis.

## TCM for Treatment of Atherosclerosis, Targeting Oxidative Stress

### Chinese Herbal Medicines and Active Metabolites of Herbs

#### Resveratrol

Resveratrol is a natural phenolic compound found in varieties of plants, such as grapes and peanut, as well as Chinese herbal medicine, including *Reynoutria japonica* Houtt [Polygonaceae; Polygoni cuspidati rhizoma et radix] and *Veratrum nigrum* L. [Melanthiaceae]. Studies revealed that resveratrol mediates anti-atherosclerosis and heart protection. It protects endothelial cells from lipid damage, promotes vasodilation by regulating nitric oxide synthesis, and scavenges oxygen radicals and superoxide radicals by limiting lipid peroxidation, inhibiting platelet aggregation, and SMCs proliferation (Mohar and Malik, 2012; Bonnefont-Rousselot, 2016; Wiciński et al., 2018). Resveratrol reduces MDA, COX-1, and Nox production and activates SOD and GSH to balance the oxidation and antioxidant capacities, leading to eNOS generation to reduce endothelial dysfunction and pathological atherosclerotic changes (Szewczuk et al., 2004; Chow et al., 2007; Vivancos and Moreno, 2008; Bruedigam et al., 2011; Li et al., 2018a; Haimei et al., 2019; Li et al., 2019c; Yang et al., 2019). Transcription factor EB (TFEB) regulates homeostasis and maintains oxidant/antioxidant balance. Resveratrol promotes the translocation of TFEB from the cytoplasm to the nucleus in human umbilical vein endothelial cells (HUVECs) to activate TFEB and exert antioxidant effects, reducing autophagy and relieving endothelial dysfunction (Zhou et al., 2019). Similar results were found in clinical trials showing a beneficial effect of resveratrol in atherosclerosis by reducing oxidative stress (Imamura et al., 2017; Mansur et al., 2017). However, Gliemann et al. revealed that, instead of heart protection, supplementation of resveratrol did not affect Sirt 1, eNOS, or SOD expression and may impair the beneficial effects of physical exercise on cardiovascular health in older men (Gliemann et al., 2013). While affirming the effect of resveratrol on atherosclerosis, some researchers raised doubts. Berbée et al. compared resveratrol with statins in the treatment of ApoE*3-Leden CETP atherosclerotic mice, and showed that resveratrol alone reduced plaque volume (similar to use atorvastatin alone), but did not affect oxidative stress-related indicators PON1, COX-1, COX2, Lox-1, and MnSOD (Berbée et al., 2013). These findings suggest that more evidence is needed to confirm resveratrol’s antioxidant effects on atherosclerosis (Table 1).

**TABLE 1:** Resveratrol for treatment of atherosclerosis by regulating oxidative stress.

Active ingredients	Subjects in study	Full botanical taxonomic names (yes/no)	Relevant gene targets	Impact on ROS related targets	Potential mechanism of AS protection	References
Resveratrol	ApoE^−/−^ mice HAECs	No	PKA-CREB	↓ROS ↑eNOS	↓endothelial dysfunction	Zhong et al. (2020)
					↓plague formation	
	High fat diet C57BL/6J mice	No	Orai1	↓peroxynitrite anion (ONOO-)	↓endothelial dysfunction	Haimei et al. (2019)
				↑eNOS		
	HUVECs	No	TyrRS-PARP1	↓MDA ↑SOD	↓endothelial damage	Yang et al. (2019)
		No	gp91^Phox^	↓Nox ↓ROS	↓ ox-LDL induced oxidative stress	Chow et al. (2007)
			rac1			
		No	TFEB	↓ROS ↓MDA	↓autophagy ↓oxidative stress	Zhou et al. (2019)
	RAW 264.7	No	—	↓ROS ↓H_2_O_2_	↓oxidative stress and inflammation	Vivancos and Moreno (2008)
				↓PGE2		
	type 2 diabetes arteriosclerosis patients	No	—	↓diacron-reactive oxygen metabolites (d-ROM)	Improve arterial stiffness in patients with T2DM	Imamura et al. (2017)
	healthy and slightly overweight volunteers	No	Sirt1	↓ROS	↑ Sirt1 ↓oxidative stress	Mansur et al. (2017)
	healthy aged physically inactive men	No	Sirt 1	No effect on eNOS, SOD, CAT, GPx-1, Nox	no effect on oxidative stress indicators	Gliemann et al. (2013)
	ApoE*3-Leiden.CETP mice	No	—	No effect on PON-1, Lox-1, MnSOD	↓ plagues volume	Berbée et al. (2013)
					no effect on oxidative stress indicators	

#### Curcumin and Its Analogues

Curcumin is the active ingredient of *Curcuma longa* L. [Zingiberaceae; Curcumae radix; Curcumae longae rhizoma]*.* It is a polyphenol demonstrated to act as a free radical scavenger and antioxidant that benefits in treating cardiovascular diseases (Zingg et al., 2013; Panahi et al., 2018; Li et al., 2019b; Li et al. 2020b). Curcumin inhibits ROS generation, limits lipid peroxidation (Panahi et al., 2018), and enhances NO bioavailability (Goel et al., 2008; Li et al., 2019a). Studies demonstrated that curcumin suppresses ROS production in both animal models and *in vitro* by inhibiting ROS-related inflammation pathways and cytokines (Zingg et al., 2013), such as ERK1/2 pathway (Ouyang et al., 2019; Zhang et al., 2020a), the high-mobility-group protein B1(HMGB1)- toll-like receptor (TLRS)-NF-κB pathway (Lv et al., 2020), COX-2 (Lee et al., 2020), and others (Shi et al., 2017; Saji et al., 2018; Asadi et al., 2020), preventing endothelial dysfunction and adhesion molecules secretion. Pu et al. found that curcumin alleviated ROS-induced endothelial dysfunction through UCP2 and increased eNOS activity (Pu et al., 2013; Treviño-Saldaña and García-Rivas, 2017). Arterial dysfunction and oxidative stress caused by vascular aging are vital factors in the development of atherosclerosis. Laboratory studies revealed some mechanisms of curcumin’s effect on aging arteries. Fleenor et al. (Fleenor et al., 2013) explored the effect of curcumin on carotid artery function and vascular oxidative stress in aged mice. They discovered that curcumin increased eNOS in the aged carotid artery, reversed endothelial-dependent dilation, reduced superoxide, and inhibited NADPH oxidase p67 subunit activity. After up-regulating ABCA1 expression and inhibiting SR-A and CD36 in macrophages, curcumin reduced ROS production and foam cell formation to stabilize vulnerable plaques (Soltani et al., 2017). Curcumin analogs, such as L3, tetrahydrocurcumin (THC), Cur-NPs(Meng et al., 2019), HASF (a dual ROS-sensitive and CD44 receptor targeting amphiphilic carrier) (Hou et al., 2020), and curcumin compounds, are synthetic compounds that have similar effects; for example, they elevate glutathione peroxidase (GPx) catalyze GSH, and have higher bioavailability than common curcumin, showing anti-oxidative stress in atherosclerosis through multiple gene targets (Naito et al., 2002; Huang et al., 2015; Zheng et al., 2016). In summary, the antioxidant effects of curcumin combat atherosclerosis by the following mechanisms: 1) Blocking enzymes that promote ROS generation; 2) increasing antioxidant enzyme activity; 3) reducing damage to endothelial cells by anti-inflammatory actions, enhancing eNOS, and reducing adhesion factors; and 4) reducing foam cell formation (more details in Table 2).

**TABLE 2:** Curcumin and its analog for treatment of atherosclerosis by regulating oxidative stress.

Active ingredients		Subjects in study		Full botanical taxonomic names (yes/no)	Relevant gene targets	Impact on ROS related targets	Potential mechanism of AS protection	References
Curcumin		VSMCs		Yes	ERK1/2	↓ROS ↓CRP	↓inflammation	Zhang et al. (2020b)
				—	TGF-β Non-smad signal pathway	↓ROS	↓oxidative stress and inflammation	Asadi et al. (2020)
		THP-1		No	ERK1/2	↓ROS	↓oxidative stress and inflammation	Ouyang et al. (2019)
					HIF-1α		↓ macrophage apoptosis	
				No	SR-A, ABCA1	↓ROS ↑GSH	↓oxidative stress and inflammation	Soltani et al. (2017)
							↓foam cell formation	
		ApoE^−/−^ mice and human cytomegalovirus (HCMV)		No	HMGB1-TLRS-NF-κB	↓ROS	↓oxidative stress and inflammation	Lv et al. (2020)
							↓endothelial dysfunction	
		HUVECs		No	PKC-CREB	↓ROS ↓COX-2	↓oxidative stress and inflammation	Lee et al. (2020)
							↓endoplasmic reticulum stress	
		Human peripheral blood mononuclear cells (HPBMCs)		No	NF-κBp65	↓ROS ↓MDA	↓oxidative stress and inflammation	Saji et al. (2018)
						↓PGE2 ↓COX		
						↓iNOS		
		human microvascular endothelial cells (HMEC)		No	NF-κB	↓ROS ↓MDA	↓oxidative stress and inflammation	Deng et al. (2017)
						↑SOD	↓adhesion molecules	
		C57BL6/N mice		No	—	↑SOD ↓Nox p67	↓endothelial dysfunction	Fleenor et al. (2013)
							↓oxidative stress	
							↓artery aging	
		SD rats		No	AMPK/UCP2	↓ROS ↑eNOS	↓endothelial dysfunction	Pu et al. (2013)
		UCP2-/- rats					↓oxidative stress	
Curcumin analogsand compounds	L3		Diabetic mice	No	—	↓ROS ↓iNOS	↓endothelial dysfunction	Zheng et al. (2016)
						↓MDA ↑SOD	↓oxidative stress	
						↑GPx ↑NO		
	compounds		THP1	No	PKCδ/Nox/ROS	↓ROS ↓Nox2	↓oxidative stress	Huang et al. (2015)
							↓matrix invasion during monocyte-macrophage differentiation	
	THC		Rabbit	No	—	↓ox-LDL	↓oxidative stress	Naito et al. (2002)

#### Salidroside

Salidroside is an active ingredient that exists in Chinese herbal medicine Hongjingtian (*Rhodiola crenulata* (Hook.f. & Thomson) H.Ohba [Crassulaceae; Rhodiolae crenulatae radix et rhizoma]); this agent reduces oxidative stress (Mao et al., 2010; Li et al., 2011a)and is used to treat atherosclerosis. In terms of relieving endothelial dysfunction after impaired ROS production, salidroside enhances eNOS production and activates several pathways, including Sirt1/Foxo1, AMPK, and Sirt3, regulating oxidative stress, inflammation, cell apoptosis, autophagy, and mitochondrial function (Xing et al., 2014; Xing et al., 2018; Zhao et al., 2019; Zhu et al., 2019b). Hemoxygenase 1(HO-1) is an important antioxidant enzyme in cell microsomes that mediates anti-inflammation and anti-oxidation and suppresses the activity of adhesion molecules (Zhang et al., 2018). Zhu et al. (Zhu et al., 2016) found that salidroside promotes the expression of HO-1 and NAD(P)H dehydrogenase-quinone oxidoreductase 1(NQO1), lowers MDA and ROS production by regulating Nrf2, and subsequently alleviates enthothelial dysfunction. Ni et al. believed that salidroside impaired the combination of ox-LDL with Lox-1 and affected foam cell formation and apoptosis in atherosclerosis by regulating Lox-1 content; during this process, there were no changes in HO-1 expression (Ni et al., 2017a). Although the anti-oxidation effect of salidroside has been determined, its specific mechanism still needs to be further explored (Table 3).

**TABLE 3:** Salidroside for treatment of atherosclerosis by regulating oxidative stress.

Active ingredients	Subjects in study	Full botanical taxonomic names (yes/no)	Relevant gene targets	Impact on ROS related targets	Potential mechanism of AS protection	References
Salidroside	Wistar rats HUVECs	No	NF-κB	↑SOD ↑NO	↓endothelial dysfunction	Xing et al. (2014)
			AMPKα	↑eNOS ↓ROS	↓Mitochondrial dysfunction	
			—	↓Superoxide anion	↓oxidative stress and inflammation	
	HUVECs	No	Nrf2	↓ROS ↓MDA	↓endothelial dysfunction	Zhu et al. (2016)
				↑SOD ↑CAT		
				↑HO-1	—	
				↑NAD(P)H dehydrogenase (quinone1) (NQO1)		
		No	AMPK,	↓ROS ↓MDA	↓oxidative stress	Zhao et al. (2019)
			SIRT1	↓Nox2 ↑SOD	↓mitochondrial dysfunction	
			—	↑GPx	↓cell apoptosis	
		No	SIRT1, FOXO1	↓ROS ↓MDA	↓oxidative stress	Zhang et at. (2020a)
				↓Nox ↑SOD	↑Autophagy	
	THP1	No	MAPK, AKT, JNK, ERK	↓Lox-1	↓foam cell formation	Ni et al. (2017a)
					↓foam cell apoptosis	
	BABLc mice	No	SIRT3	↑eNOS	↓oxidative stress induced premature senescence	Xing et al. (2018)
	HUVECs				↓inflammation	

#### Active Ingredients of Danshen

The traditional Chinese herbal medicine Danshen (*Salvia miltiorrhiza* Bunge [*Lamiaceae*; *Salviae miltiorrhizae* radix et rhizoma]) has been widely applied in the treatment of atherosclerotic diseases in Asia with encouraging results (Li et al., 2018b; Ren et al., 2019). The chemical components in *Salvia* are divided into fat-soluble and water-soluble components. Salvianolic acids (A-G) are water-soluble components. Tanshinone I, tanshinone IIA, tanshinone IIB, methyl tanshinate, and danshendiol are fat-soluble components. Investigators discovered that salvianolic acids have a good therapeutic effect on atherosclerosis (Xiang et al., 2018; Ma et al., 2020; Yang et al., 2020b). In a diabetic rat model, salvianolic acid B (Ren et al., 2016) decreased Nox2 and Nox4 expression, limiting ROS that caused endothelial dysfunction. The vasoprotective factors eNOS and NO also improved in this process, preventing the occurrence of atherosclerosis. Tanshinone IIA is one of the pharmacologically active ingredients derived from Danshen that participates in maintaining vascular homeostasis; it is widely used to prevent and treat coronary heart disease (CHD) in Asia (Gao et al., 2012; Feng et al., 2021). Tanshinone IIA competes with LDL oxidation induced by diverse oxidizing systems, like copper-, peroxyl radical-, and peroxynitrite, scavenging peroxyl radicals and preventing atherosclerosis (Niu et al., 2000). In animal experiments (the ApoE^−/−^ model and high fatty diet rabbit), tanshinone IIA exerted antioxidant and anti-inflammatory effects by reducing ox-LDL, ROS, MDA, and other pro-oxidative stress products to reduce lipid peroxidation and inhibit atherosclerosis progression via multiple targets (ERK, NF-κB, PPARγ, and miR) (Fang et al., 2008; Tang et al., 2011; Chen et al., 2012; Liu et al., 2015; Xuan et al., 2017). *In vitro* experiments showed that tanshinone IIA reduced the damage caused by H_2_O_2_, increased the production of antioxidant enzymes, and prevented endothelial cell injury from oxidative stress (Lin et al., 2006; Zhu et al., 2017a). Adjusting antioxidant enzyme GPx generation may be a critical step in reducing ROS-related functions like apoptosis, endothelial injury, and inflammation (Li et al., 2008; Fang et al., 2008; Zhu et al., 2017a). Tanshinone IIA also alleviates ROS-induced subsequent autophagy and apoptosis (Ni et al., 2017b; Chen et al., 2012; Huimin et al., 2017; Li et al., 2008), presenting potential anti-atherosclerosis effects (Table 4).

**TABLE 4:** Active ingredients in Danshen for treatment of atherosclerosis by regulating oxidative stress.

Active ingredients	Subjects in study	Full botanical taxonomic names (yes/no)	Relevant gene targets	Impact on ROS related targets	Potential mechanism of AS protection	References
Salvianolic acid B	diabetic rat	No	Bcl-2	↓MDA ↑SOD	↓oxidative stress	Ren et al. (2016)
				↑eNOS↑NO	↓endothelial dysfunction	
				↓Nox2↓Nox4		
Tanshinone IIA	LDL solution	Yes	—	↑SOD	↓LDL oxidation	Niu et al. (2000)
				↓ONOO-	↓oxidative stress	
	ApoE^−/-^ mice	Yes	miR-146b	↓ox-LDL	↓oxidative stress and inflammation	Xuan et al. (2017)
			miR-155			
		No	ERK, NF-κB	↓MDA ↑SOD	↓oxidative stress and inflammation	Liu et al. (2015)
	ApoE^−/-^ mice Macrophages of mice	Yes	PPARγ	↓SR-A ↓ox-LDL	↓oxidative stress	Tang et al. (2011)
	rabbit	Yes	—	↓ROS ↓MDA	↓oxidative stress and inflammation	Fang et al. (2008)
		No		↓ox-LDL		Chen et al. (2012)
				↑SOD ↑GPx		
	HUVECs	Yes	Pregnane X receptor (PxR)	↑GSH disulfide	↓Mitochondrial Apoptosis	Zhu et al. (2017a)
					↓endothelial injury	
	HUVECs	No	—	↑SOD ↑NO	↓oxidative stress and inflammation	Lin et al. (2006)
					↓endothelial injury	
	J774 macrophage	No	—	↑GPx	↓apoptosis	Li et al. (2008)
					↓oxidative stress	
	HUVECs	No	PI3K/Akt/mTOR?LC3-Ⅰ?LC3-Ⅱ	↓MDA ↑SOD	↓oxidative stress	Huimin et al. (2017)
					↓autophagy	

#### Berberine

Berberine is an active ingredient derived from the Chinese herbal medicine *Coptis chinensis* Franch. [Ranunculaceae; Coptidis rhizoma] that helps to treat atherosclerosis-related cardiovascular diseases (Mulvihill and Huff, 2010; Wu et al., 2010). It inhibits endothelial cell dysfunction via multiple mechanisms (Fatahian et al., 2020). By regulating UCP2 and Nox2, berberine blocks MDA and enhances SOD production, inhibiting the AMPK pathway to relieve inflammation and autophagy; it also protects against endothelial cell dysfunction in atherosclerosis (Sarna et al., 2010; Wang et al., 2011; Fan et al., 2015; Caliceti et al., 2017; Tan et al., 2020). Accumulation of foam cells and apoptosis leads to increased lipid core volume and thinning of the fibrous caps, resulting in rupture of vulnerable plaques. Studies showed that berberine maintained lipid homeostasis and reduced foam cells formation via LXRα/ABCA1 and Nrf/HO-1, limiting macrophage ox-LDL uptake and cholesterol efflux and inhibiting macrophages superoxide anion production (Lee et al., 2010; Yang et al., 2020c). Berberine also decreased endothelin-1 (ET-1) induced Lox-1 expression in monocyte-derived macrophages, acting as an antioxidant and reducing foam cell formation (Chi et al., 2014; Caliceti et al., 2017). Zhu X et al. (Zhu et al., 2017b) established a premature aging model using low-concentration berberine to interfere with hydrogen peroxide; they found that berberine combated premature aging in human diploid fibroblasts via Sirt1-mediated ROS reduction, protecting loss of mitochondrial membrane potential and showing an antioxidant effect. Berberine may inhibit cell senescence caused by reducing oxidative stress associated with age-related diseases. Berberine also inhibited the proliferation and migration of VSMCs by suppressing Nox activity (Cho et al., 2005) and suppressed ROS-dependent NLRP3 inflammasomes in human peripheral blood mononuclear cells (PBMCs) (Jiang et al., 2017), providing research targets for berberine in treating atherosclerosis. (Table 5).

**TABLE 5:** Berberine for treatment of atherosclerosis by regulating oxidative stress.

Active ingredients	Subject in study	Full botanical taxonomic names (yes/no)	Relevant gene targets	Impact on ROS related targets	Potential mechanism of AS protection	References
Berberine	ApoE-/- mice AMPKα2^⁻/⁻^/ApoE^−/-^mice HUVECs	No	UCP2	↓MDA ↓ROS	↓endothelial injury	Wang et al. (2011); Tan et al. (2020)
			AMPK	↓IL-6	↓oxidative stress	
	J774A.1	No	AMPK/mTOR	—	↓ox-LDL induced inflammation	Fan et al. (2015)
					↓autophagy	
	THP-1	No	NF-κB	↓Nox	↓oxidative stress and inflammation	Jiang et al. (2017)
			NLRP3			
		No	NADPHgp91(Nox2 subunit)	↑SOD	↓endothelial dysfunction	Sarna et al. (2010)
		No	LXRα	—	regulate lipid homeostasis	Lee et al. (2010)
			ABCA1		↓ox-LDL induced lipid accumulation	
	ApoE-/- mice THP-1	No	Nrf2/HO-1		↓foam cell formation	Yang et al. (2020c)
	Fibroblast cell 2BS	No	sirt1	↓ROS	↓oxidative stress	Zhu et al. (2017b)
					↓H_2_O_2_ induced Damage of mitochondrial membrane	
	SD rats Monocyte-derived macrophages (MDM)	No	ET-1	↓Lox-1 ↓MDA	↓oxidative stress	Chi et al. (2014)
				↑SOD	↓foam cell formation	
	HUVECs	No	TNFα	↓ROS↓Lox-1 ↓Nox2	↓oxidative stress and inflammation.	Caliceti et al. (2017)
			NF-κB		↓endothelial dysfunction	
			MAPK/Erk1/2			
	VSMCs	No	ERK1/2	↓ROS	↓VSMCs proliferation and migration	Cho et al. (2005)

#### Quercetin

Quercetin is widely distributed in the plant kingdom. Chinese herbal medicines such as *Styphnolobium japonicum* (L.) Schott [Fabaceae; Sophorae flos], *Platycladus orientalis* (L.) Franco [Cupressaceae; Platycladi cacumen], *Alpinia officinarum* Hance [Zingiberaceae; Alpiniae officinarum rhizoma], *Tussilago farfara* L. [Asteraceae; Farfarae flos], *Morus alba* L. [Moraceae; Mori folium; Mori cortex], and *Ginkgo biloba* L. [Ginkgoaceae; Ginkgo folium; Ginkgo semen] (Zhanxia et al., 2019) are major sources of quercetin. Quercetin is also found in fruits, nuts, and vegetables (Stromsnes et al., 2020). Studies showed that quercetin has potential to treat atherosclerosis by reducing inflammation and resisting oxidative stress (Deng et al., 2020). The antioxidant effects of quercetin occur first by inhibition of p47phox and p67phox activity, reducing NADPH oxidase activation to decrease ROS production (Xiao et al., 2017). Sirt1 and AMPK may be potential key targets for reducing Nox2 and Nox4 expression (Hung et al., 2015) and regulating ROS, superoxide (Loke et al., 2010), and MDA production (Yi et al., 2011; Lara-Guzman et al., 2012). Quercetin also increases generation of antioxidants such as HO-1 (Shen et al. 2013; Luo et al., 2020), NAD(P)H dehydrogenase, and glutamate-cysteine ligase (GCL) by activating Nrf2 (Li et al., 2016b). Li et al. (Li et al., 2016a) studied the mechanism of quercetin restoring the expression of GSH in human aortic endothelial cells (HAEC) that was relevant to GCL. In this manner, quercetin inhibited oxidative stress. After balancing oxidation and anti-oxidation, NO and eNOS bioavailability improved, reducing inflammation (Lara-Guzman et al., 2012; Bhaskar et al., 2016; Lu et al., 2017; Xue et al., 2017; Jia et al., 2019), autophagy (Cao et al., 2019), and apoptosis (Lu et al., 2017; Jiang et al., 2020), and inhibiting the recruitment of monocytes by adhesion factors, thereby protecting endothelial cell function. By elevating HDL cholesterol absorption capacity, quercetin increased HDL anti-oxidation and reduced lipid accumulation (Lara-Guzman et al., 2012; Cui et al., 2017; Xue et al., 2017; Jia et al., 2019) (Table 6).

**TABLE 6:** Quercetin for treatment of atherosclerosis by regulating oxidative stress.

Active ingredients	Subjects in study	Full botanical taxonomic names (yes/no)	Relevant gene targets	Impact on ROS related targets	Potential mechanism of AS protection	References
Quercetin	ApoE^−/-^ mice mouse peritoneal macrophages (MPMs)	no	—	↓ROS ↓p47phox	↓oxidative stress	Xiao et al. (2017)
				↓p67phox		
	ApoE-/- mice HUVECs	no		↓ROS ↓p47phox	↓oxidative stress	Luo et al. (2020)
				↑NO ↑HO-1	↓endothelial dysfunction	
	ApoE-/- mice HAEC	no		↑eNOS ↑HO-1	↓endothelial dysfunction	Shen et al. (2013)
	HAEC	no		↑GSH	↓oxidant production	Li et al. (2016a)
	HUVECs	no	p38	↓oxidant production	↓endothelial damage	Li et al. (2016b)
			Nrf2	↑HO-1 ↑GCL		
		no	SIRT1 AMPK	↓Nox2 ↓Nox4	↓oxidative stress	Hung et al. (2015)
			NF-κB		↓endothelial dysfunction	
		no	—	↓MDA ↑NO	↓oxidative stress	Yi et al. (2011)
					↓endothelial dysfunction	
	ApoE-/- mice bone marrow-derived macrophages	no	CD36	↓MDA	↓oxidative stress and inflammation	Loke et al. (2010); Lara-Guzman et al. (2012)
			gp91^Phox^	↓Vascular superoxide	↓foam cell formation	
			rac1	↑HO-1 ↑eNOS	↓endothelial dysfunction	
		no	Sirt1	↓ROS	↓oxidative stress	Jiang et al. (2020)
					↓apoptosis	
		no	PCSK9 CD36	↓ox-LDL	↓oxidative stress and inflammation	Jia et al. (2019)
			PPARγ LXRα		↓lipid deposition	
		no	—	↓MDA ↓oxidized phosphocholine	↓HDL oxidation	Cui et al. (2017)
	RAW264.7	no	—	↓ROS ↓Lox-1	↓oxidative stress and inflammation	Xue et al., (2017)
					↓lipid deposition	
	C57BL/6	no	PI3K/AKT	↓ROS	↓oxidative stress and inflammation	Lu et al. (2017)
	VSMCs		NF-κB		↓apoptosis	
	HUVECs	no	TLR/NF-κB	↓MPO ↓COX	↓inflammation	Bhaskar et al. (2016)
					↓endothelial dysfunction	
	RAW264.7	no	MST1 LC3-II/I	↓ROS	↓autophagy	Cao et al. (2019)
			SA-β-GAL		↓foam cell formation	
			—		↓aging	

#### Other Active Ingredients of Herbs

The Chinese herbal medicine Sanqi (*Panax notoginseng* (Burkill) F.H.Chen [*Araliaceae*; Notoginseng radix et rhizoma]) is a traditional medicine widely used to treat CHD in China. It was shown to have good efficacy and safety in clinical practice (Sun et al., 2016; Duan et al., 2017). Panax notoginseng saponins, including ginsenoside Rb1, ginsenoside Rg1, and notoginsenoside R1, are the active ingredients extracted from Sanqi; they reduce ROS generation by inhibiting NOX4 activity and block recruitment of adhesion molecules to monocytes induced by multiple pathways (Dou et al., 2012; Qiao et al., 2015; Fan et al., 2016), protecting endothelial function and preventing atherosclerosis. Ginsenoside, derived from the Chinese herbal medicine *Panax ginseng* C.A.Mey. [*Araliaceae*; Ginseng radix et rhizoma], includes ginsenosides Rg1 and Rb1 (also abstracted from Sanqi), Rb2, Rb3, Rg2, Rg3, Rf, F1, F2, Rd, and Rh2 (Jin et al., 2019). Lü et al. (Lü et al., 2019) found that Rb1 competitively inhibited the expression of the estrogen receptor ER-β, reducing ROS generation in endothelial cells and increasing eNOS and SOD, thereby reducing endothelial dysfunction. Coupled with ROS reduction, the inflammatory response was also suppressed to alleviate atherosclerosis (Fan et al., 2016; Zhou et al., 2017). Similar results were also found in ginsenoside F1, which reduced LDL-induced endothelial dysfunction; it may be considered a new medication to treat atherosclerosis (Li et al., 2011b; Qin et al., 2017). *Ginko biloba* L. [Ginkgoaceae; Ginkgo folium; Ginkgo semen]is a dioecious tree species native to China. Flavonoids and terpenoids are the primary active compounds in *Ginkgo biloba* leaves. They have various pharmacological effects, including anti-oxidation, anti-platelet, and anti-apoptosis, preventing and treating cardiovascular and cerebrovascular diseases, Alzheimer’s disease, and atherosclerosis (Li et al., 2020c; Tian et al., 2017). *Ginkgo biloba* extract (GBE) is used in modern medicine. The standard GBE- EGB761 synthesized by Willmar Schwabe Pharmaceuticals includes terpenoids, flavonoids, alkylphenols, polypentanol, and organic acids (van Beek and Montoro, 2009). To reduce ROS, GBE inhibits NADPH oxidase subunits p47 (Phox) and rac-1; it also reduces gp91 and p22 (Phox) expression caused by ox-LDL induced AMPK and PKC activation (Ou et al., 2013). GBE also enhances HO-1 expression through the Akt/eNOS and p38/MAPK pathways (Tsai et al., 2013). It reduces the adhesion molecules such as monocyte chemokine-1 (MCP-1) and VCAM-1 mediated by ROS and prevents the adhesion of monocytes to endothelial cells, protecting endothelial cells’ function (Chen et al., 2003; Ou et al., 2009; Piazza et al., 2019). In terms of inhibiting the formation of foam cells, Li et al. (Tonghua, 2019) found that EGb761 inhibited the uptake of cholesterol by VSMC smooth muscle cells and enhanced the efflux of cholesterol by smooth muscle cells. EGb761 treatment inhibited the expression of SR-A1 and LOX-1, thereby inhibiting the uptake of ox-LDL by smooth muscle cells. Ginkgolide B, another active component abstracted from *Ginkgo biloba* leaves, presented similar mechanisms to EGB761 (Li et al., 2009; Ma et al., 2013; Feng et al., 2018; Wang et al., 2019). The antioxidant effect of GBE on atherosclerosis is mediated by reducing ROS generation, thereby preventing endothelial dysfunction caused by the adhesion of monocytes and endothelial cells (Jung et al., 2012) (Table 7).

**TABLE 7:** Other active ingredients in herbs that treat atherosclerosis by regulating oxidative stress.

Active ingredients	Subjects in study	Full botanical taxonomic names (yes/no)	Relevant gene targets	Impact on ROS related targets	Potential mechanism of AS protection	References
Panax- notoginseng saponins	ApoE^−/-^ mice	No	RAGE/MAPK	↓Nox4	↓oxidative stress and inflammation	Dou et al. (2012)
		Yes	NF-κB	↓ROS ↓MDA	↓endothelial dysfunction	Qiao et al. (2015)
		Yes	Nrf2	↑SOD ↑GSH	↓adhesion molecules expression and adhesion to monocytes	Fan et al. (2016)
			TNF-α-p38	↑HO-1		
Ginsenoside Rb1	ApoE^−/-^ mice HUVECs	No	JNK	↓ROS↓MDA	↓oxidative stress and inflammation	Zhou et al. (2017); Lü et al. (2019)
			TNF-α	↓GPx ↑SOD	↓oxidative stress	
			NF-κB	↑CAT ↑eNOS	↓endothelial dysfunction	
			ER-β	↓Superoxide anion		
			—	↑eNOS		
Ginsenoside F1	ApoE^−/-^ mice	Yes	NF-κB	↓MPO ↓Lox-1	↓endothelial dysfunction	Qin et al. (2017)
					↓inflammation	
EGB761	HUVECs	No	AMPK PKC NF-κB	↓p47 (phox) ↓ Rac-1 ↓gp91 (phox) ↓p22 (phox) ↓ROS ↓Lox-1 ↑HO-1 ↓MCP-1 ↓VCAM-1 ↓ ICAM ↓E-selectin	↓endothelial dysfunction ↓inflammation ↓oxidative stress ↓adhesion molecules expression and adhesion to monocytes ↓foam cells formation	Ou et al. (2013) Tsai et al. (2013); Ou et al. (2009); Chen et al. (2003); Tonghua (2019)
	HAECs					
	VSMCs					
Ginkgolide B	HUVECs	No	PCSK-9 LDL-R sirt1 Akt Nrf2	↓Lox-1 ↓Nox4 ↓MCP-1 ↓ROS ↓VCAM-1 ↓ ICAM ↓E-selectin ↓inflammatory factors	↓endothelial dysfunction ↓inflammation ↓oxidative stress ↓adhesion molecules expression and adhesion to monocytes	Feng et al. (2018); Li et al. (2009); Ma et al. (2013); Wang et al. (2019)

### TCM Preparations and Related Drugs

TCM preparations and its patent drugs include many Chinese herbs containing various ingredients. Clinical studies and laboratory research have shown positive effects on oxidative stress in atherosclerosis-related diseases (Hanqing et al., 2018; Feng et al., 2019; Xibin et al., 2019; Yonggang, 2020; Zhong et al., 2020). Tongqiao Huoxue decoction and Yiqi Huoxue decoction reduce MDA and ox-LDL production in ischemic stroke patients, relieve oxidative stress, and improve cerebral blood flow (Yonggang, 2020; Liu and Cui, 2016). Liang et al. (Hao et al., 2019) found that combined use of acupuncture and Dan-Lou tablets in patients with hypertension and atherosclerosis inhibited ROS production, enhanced SOD, and reduced inflammatory factors. Huatan Quzhuo fang exerted antioxidant effects in carotid atherosclerotic plaque patients (Quan et al., 2019). Dachaihu decoction (Gexuan et al., 2019) and Buyang Huanwu decoction (Zhixin et al., 2019) may reduce angina pectoris frequency in CHD patients through increasing SOD, total antioxidant capacity, and other antioxidant indicators. Xuefu Zhuyu decoction, which is frequently applied to treat blood stasis diseases in China, also shows alleviated atherosclerosis (Xibin et al., 2019; Feng et al., 2019; Zhao et al., 2020a), revealing a protective effect on oxidative stress (Table 8). According to Liu et al. (Liu et al., 2020), Buyang Huanwu decoction regulated oxidative stress and inflammation through TGF-β and NF-κB pathways, reducing MDA production and increasing CAT expression in a rat model of atherosclerosis. And the antioxidant effect achieved by Gualou Xiebai Banxia decoction was the inhibition of Lox-1 in aorta and enhancement of SOD and GPx generation (Jianen et al., 2017). Similar findings were also observed in Chinese medicine patent medications as well (Yu et al., 2016; Lanbin et al., 2017; Tong et al., 2018; Cailian and Zhang, 2019; Shibai et al., 2019; Yufeng et al., 2019; Zhu et al., 2019c; Yansheng et al., 2020). In CHD patients, clinical studies revealed that *Salvia miltiorrhiza* polyphenolate downregulated endothelin-1 expression, reducing the frequency of angina pectoris and improving heart function by protecting endothelial function and moderating oxidative stress (Jingshu et al., 2019; Feilong et al., 2020) (more details in Table 8 and Table 9, raw herbs of each preparation listed in Table 10).

**TABLE 8:** TCM preparations that treat atherosclerosis by regulating oxidative stress.

Preparations	Subjects in study	Full botanical taxonomic names (yes/no)	Impact on ROS related targets	Potential mechanism in treating atherosclerotic diseases	Single (1) or combined with basic treatment (2)	References
Tongqiao Huoxue decoction	acute ischemic stroke patients	No	↓MDA ↓ox-LDL	↓oxidative stress	(2)	Yonggang (2020)
			↑SOD ↑GPx			
Yiqi Huoxue decoction	ischemic stroke patients	No	↓MDA ↓ox-LDL	↓oxidative stress	(2)	Liu and Cui (2016)
			↑SOD ↑GPx			
Yangmai Huatan decoction	Hypertension with atherosclerosis patients	No	↓MDA ↑SOD	↓oxidative stress and inflammation	(2)	Hao et al. (2019)
Huatan Quzhuo fang	carotid atherosclerotic plaque patients	No	↓MDA ↑SOD	↓oxidative stress	(2)	Quan et al. (2019)
				↑lipid regulation		
Dachaihu decoction	unstable angina patients	No	↓MDA ↓LPO	↓endothelial dysfunction	(2)	Gexuan et al. (2019)
			↑SOD ↑eNOS	↓oxidative stress		
			↑TAC			
Buyang Huanwu decoction	unstable angina patients	No	↑SOD ↑GSH	↓oxidative stress	(2)	Zhixin et al. (2019)
			↑TAC ↑NOS			
	SD rats	No	↓MDA ↑SOD	↓oxidative stress and inflammation	(1)	Liu et al. (2020)
			↑GPx ↑CAT			
Xuefu Zhuyu decoction	PCI patients	No	↓MDA ↑SOD	—	(2)	Zhao et al. (2020b)
	SD rats	No	↓ROS ↓Nox2		(1)	Xibin et al. (2019)
	domestic rabbits	No	↓ROS ↓MDA		(2)	Hanqing et al. (2018)
			↓Nox2 ↑TAC			
			↑SOD ↑GSH			
	acute cerebral infarction patients	No	↓MDA ↑SOD	↓oxidative stress	(2)	Feng et al. (2019)
			↑NO	↓endothelial dysfunction		
Gualou Xiebai Banxia decoction	ApoE^−/-^ mice	No	↓MDA ↓ox-LDL	↓oxidative stress	(1)	Jianen et al. (2017)
			↑SOD ↑GPx			
Huotan Jiedu Tongluo decoction	Japanese white rabbit	No	↓ROS ↓ox-LDL	↓oxidative stress	(1)	Tong et al. (2018)
			↓eNOS uncoupling			
Dingxin fang	ApoE^−/-^ mice	No	↓MDA ↓ox-LDL	↓oxidative stress	(1)	Yu et al. (2016)
			↑SOD ↑GPx			
			↑TAC			
Huanglian Jiedu decoction	SD rats	No	↓MDA ↓ox-LDL	↓oxidative stress and inflammation	(1)	Lanbin et al. (2017)
			↑SOD			

**TABLE 9:** Patent drugs that treat atherosclerosis by regulating oxidative stress.

Patent drugs	Subjects in study	Full botanical taxonomic names (yes/no)	Impact on ROS related targets	Potential mechanism in treating atherosclerotic diseases	Single (1) or combined with basic treatment (2)	References
Danshen granules, capsules, tablets, and drop pills	unstable angina patients	no	↓MDA ↑SOD	↓oxidative stress and inflammation	(2)	Yansheng et al. (2020)
			↑GPx ↑TAC			
Shexiang Baoxin pills	post PCI patients	no	↓MDA ↓LPO	↓oxidative stress	(2)	Yufeng et al. (2019)
			↑SOD ↑TAC			
Qishen Yiqi drip pills	coronary heart disease	no	↓MDA ↑SOD	↓oxidative stress	(2)	Cailian and Zhang (2019)
Dan-Lou tablet	Wistar rats	no	↓MDA ↓ox-LDL	↓oxidative stress and inflammation	(1)	Zhu et al. (2019c)
			↑SOD			
Ginkgo Leaf Capsules	angina pectoris patients	no	↓MDA ↑SOD	↓oxidative stress and inflammation	(2)	Shibai et al. (2019)
			↑TAC			
Salvia miltiorrhiza polyphenolate	CAD patients	no	↓MDA ↑SOD	↓oxidative stress	(2)	Jingshu et al. (2019)
			↑NO	↓endothelial dysfunction		
	CAD and angina pectoris patients	no	↓LPO ↓MDA	↓oxidative stress	(2)	Feilong et al. (2020)
			↑SOD ↑total anti-oxidative capacity (TAC)			

**TABLE 10:** Raw herbs in TCM preparations.

Preparations	Raw herbs
Tongqiao Huoxue decoction	*Prunus persica* (L.) Batsch [Rosaceae; Persicae semen], *Carthamus tinctorius* L. [Asteraceae; Carthami Flos], *Zingiber officinale* Roscoe [Zingiberaceae; Zingiberis rhizoma praeparatum], *Paeonia lactiflora* Pall. [Paeoniaceae; Paeoniae radix alba], *Conioselinum anthriscoides 'Chuanxiong'* [Apiaceae; Chuanxiong rhizoma], *Ziziphus jujuba* Mill. [Rhamnaceae; Jujubae fructus]
Yiqi Huoxue decoction	*Astragalus mongholicus* Bunge [Fabaceae; Astragali radix], *Salvia miltiorrhiza* Bunge [Lamiaceae; Salviae miltiorrhizae radix et rhizoma], *Paeonia lactiflora* Pall. [Paeoniaceae; Paeoniae radix alba], *Angelica sinensis* (Oliv.) Diels [Apiaceae; Angelicae sinensis radix], *Conioselinum anthriscoides 'Chuanxiong'* [Apiaceae; Chuanxiong rhizoma], *Achyranthes bidentata* Blume [Amaranthaceae; Achyranthis bidentatae radix], *Dioscorea oppositifolia* L. [Dioscoreaceae; Dioscoreae rhizoma], *Prunus persica* (L.) Batsch [Rosaceae; Persicae semen], *Spatholobus suberectus* Dunn [Fabaceae; Spatholobi caulis], *Rehmannia glutinosa* (Gaertn.) DC. [Orobanchaceae; Rehmanniae radix], *Carthamus tinctorius* L. [Asteraceae; Carthami Flos], *Glycyrrhiza glabra* L. [Fabaceae; Glycyrrhizae radix et rhizoma]
Yangmai Huatan decoction	*Astragalus mongholicus* Bunge [Fabaceae; Astragali radix], *Smilax glabra* Roxb. [Smilacaceae; Smilacis glabrae rhizoma], *Salvia miltiorrhiza* Bunge [Lamiaceae; Salviae miltiorrhizae radix et rhizoma], *Carthamus tinctorius* L. [Asteraceae; Carthami Flos], *Prunus persica* (L.) Batsch [Rosaceae; Persicae semen], *Pinellia ternata* (Thunb.) Makino [Araceae; Pinelliae rhizoma], *Atractylodes macrocephala* Koidz. [Asteraceae; Atractylodis macrocephalae rhizoma], *Wurfbainia villosa* (Lour.) Skornick. & A.D.Poulsen [Zingiberaceae; Amomi fructus], *Gastrodia elata* Blume [Orchidaceae; Gastrodiae rhizoma], *Crataegus pinnatifida* Bunge [Rosaceae; Crataegi fructus], *Hordeum vulgare* L. [Poaceae; Hordei fructus germinatus]
Huatan Quzhuo fang	*Panax ginseng* C.A.Mey. [Araliaceae; Ginseng radix et rhizoma], *Atractylodes macrocephala* Koidz. [Asteraceae; Atractylodis macrocephalae rhizoma]*, Pinellia ternata* (Thunb.) Makino [Araceae; Pinelliae rhizoma]*, Smilax glabra* Roxb. [Smilacaceae; Smilacis glabrae rhizoma]*, Crataegus pinnatifida* Bunge [Rosaceae; Crataegi fructus]*, Citrus × aurantium* L. [Rutaceae; Citri exocarpium rubrum], *Citrus × aurantium* L. [Rutaceae; Aurantii fructus immaturus], *Nelumbo nucifera* Gaertn. [Nelumbonaceae; Nelumbinis folium], *Alisma plantago-aquatica subsp. orientale* (Sam.) Sam. [Alismataceae; Alismatis rhizoma]*, Reynoutria multiflora* (Thunb.) Moldenke [Polygonaceae; Polygoni multiflori radix]*, Salvia miltiorrhiza* Bunge [Lamiaceae; Salviae miltiorrhizae radix et rhizoma]
Dachaihu decoction	*Bupleurum chinense* DC. [Apiaceae; Bupleuri radix], *Citrus × aurantium* L. [Rutaceae; Aurantii fructus immaturus], *Scutellaria baicalensis* Georgi [Lamiaceae; Scutellariae radix], *Paeonia lactiflora* Pall. [Paeoniaceae; Paeoniae radix alba], *Salvia miltiorrhiza* Bunge [Lamiaceae; Salviae miltiorrhizae radix et rhizoma], *Smilax glabra* Roxb. [Smilacaceae; Smilacis glabrae rhizoma], *Citrus × aurantium* L. [Rutaceae; Citri exocarpium rubrum], *Rheum officinale* Baill. [Polygonaceae; Rhei radix et rhizoma], *Pinellia ternata* (Thunb.) Makino [Araceae; Pinelliae rhizoma], *Glycyrrhiza glabra* L. [Fabaceae; Glycyrrhizae radix et rhizoma]
Buyang Huanwu decoction	*Astragalus mongholicus* Bunge [Fabaceae; astragali radix], *Angelica sinensis* (Oliv.) Diels [Apiaceae; angelicae sinensis radix], *Paeonia lactiflora* Pall. [Paeoniaceae; paeoniae radix alba], *Rehmannia glutinosa* (Gaertn.) DC. [Orobanchaceae; rehmanniae radix], *Conioselinum anthriscoides 'Chuanxiong'* [Apiaceae; chuanxiong rhizoma], *Prunus persica* (L.) Batsch [Rosaceae; persicae semen], *Carthamus tinctorius* L. [Asteraceae; Carthami Flos]
Xuefu Zhuyu decoction	*Prunus persica* (L.) Batsch [Rosaceae; Persicae semen], *Carthamus tinctorius* L. [Asteraceae; Carthami Flos]*, Angelica sinensis* (Oliv.) Diels [Apiaceae; Angelicae sinensis radix]*, Bupleurum chinense* DC. [Apiaceae; Bupleuri radix]*, Rehmannia glutinosa* (Gaertn.) DC. [Orobanchaceae; Rehmanniae radix]*, Citrus × aurantium* L. [Rutaceae; Citri reticulatae pericarpium], *Conioselinum anthriscoides 'Chuanxiong'* [Apiaceae; Chuanxiong rhizoma]*, Paeonia lactiflora* Pall. [Paeoniaceae; Paeoniae radix alba]*, Platycodon grandiflorus* (Jacq.) A.DC. [Campanulaceae; Platycodonis radix], *Glycyrrhiza glabra* L. [Fabaceae; Glycyrrhizae radix et rhizoma]*, Achyranthes bidentata* Blume [Amaranthaceae; Achyranthis bidentatae radix]
Gualou Xiebai Banxia decoction	*Trichosanthes kirilowii* Maxim. [Cucurbitaceae; Trichosanthis fructus], *Allium chinense* G.Don [Amaryllidaceae; Allii macrostemonis bulbus], *Pinellia ternata* (Thunb.) Makino [Araceae; Pinelliae rhizoma]
Huotan Jiedu Tongluo decoction	*Lonicera japonica* Thunb. [Caprifoliaceae; Caulis lonicerae japonicae], *Angelica sinensis* (Oliv.) Diels [Apiaceae; Angelicae sinensis radix]*, Trichosanthes kirilowii* Maxim. [Cucurbitaceae; Trichosanthis fructus]
Dingxin fang	*Salvia miltiorrhiza* Bunge [Lamiaceae; Salviae miltiorrhizae radix et rhizoma]*, Panax notoginseng* (Burkill) F.H.Chen [Araliaceae; Notoginseng radix et rhizoma]*, Sophora flavescens* Aiton [Fabaceae; Sophorae flavescentis radix], *Coptis chinensis* Franch. [Ranunculaceae, Coptidis rhizoma], *Ziziphus jujuba* Mill. [Rhamnaceae; Ziziphi Spinosae Semen], *Codonopsis pilosula* (Franch.) Nannf. [Campanulaceae; Codonopsis radix]*, Trichosanthes kirilowii* Maxim. [Cucurbitaceae; Trichosanthis fructus], *Paeonia lactiflora* Pall. [Paeoniaceae; Paeoniae radix alba], *Smilax glabra* Roxb. [Smilacaceae; Smilacis glabrae rhizoma]
Huanglian Jiedu decoction	*Coptis chinensis* Franch. [Ranunculaceae, Coptidis rhizoma]*, Scutellaria baicalensis* Georgi [Lamiaceae; Scutellariae radix]*, Phellodendron chinense* C.K.Schneid. [Rutaceae; Phellodendri chinensis cortex]*, Gardenia jasminoides* J.Ellis [Rubiaceae; Gardeniae fructus]

## Discussion

The application of antioxidants in the treatment of atherosclerosis are still under exploration. Clinical studies showed that natural antioxidants such as vitamin C and vitamin E did not reduce cardiovascular events (Libby et al., 2011). ACE inhibitors, ARB, aspirin, and statins reduce ROS generation and improve antioxidant effects (Kattoor et al., 2017). However, clear evidence of atherosclerotic antioxidant effects of these drugs remains insufficient. Probucol is a synthetic antioxidant used to regulate lipids and treat atherosclerosis. Kim et al. (Joon et al., 2018) found that combination use of aspirin or cilostazol with probucol can reduce vascular events in ischemic stroke at high risk of cerebral hemorrhage patients; however, they failed to demonstrate decreased risk of myocardial infarction. AGI-1067, an equivalent antioxidant and modifier of probucol, reduced restenosis after PCI without prolonging the QTc interval (Tardif et al., 2003). In a randomized double-blind placebo trial of acute coronary syndrome, AGI-1067 reduced composite secondary endpoint events such as primary outcome with all deaths, cardiovascular death, non-fatal myocardial infarction, or non-fatal stroke. Nevertheless, the agent insufficiently reduced the primary endpoint events, including cardiovascular death, myocardial infarction (non-fatal), stroke (non-fatal), and unstable angina, and was more likely to cause adverse events such as anemia and bleeding (Tardif et al., 2008). Further evaluation is required before the drug is officially used in the clinical treatment of atherosclerosis.

Chinese herbal medicine has a history spanning thousands of years and has been widely used to treat atherosclerosis in China. Based on our summary, current evidence from the studies illustrates that Chinese herbal medicines, herbal active metabolites, and TCM preparations have made progress in the antioxidant-mediated treatment of atherosclerosis. And the underlying mechanisms of these ingredients are also elucidated in a more specific manner, such as preventing plaque progression through protecting endothelial function, lipid metabolism, and foam cell formation. Researchers provided experimental bases for and clinical verifications of antioxidant targets of TCM. These ingredients may serve as alternatives for treatment of atherosclerosis via management of oxidative stress.

Nevertheless, there are limitations and controversies that hinder the promotion of these results. Most studies concluded in this review failed in stating the source plants of the active ingredients in appropriate botanical nomenclature (shown in Tables 1–8 and Table 10). Ambiguous or incorrect use of botanical nomenclature may hinder the accuracy and promotion of research results since readers may not recognize which plants are being referenced (Rivera et al., 2014).

Most clinical studies have an insufficient number of patients and a lack of large-scale multi-center clinical studies. TCM preparations consist of various Chinese herbs that may involve multiple chemical components. Their complexity and diversity creates challenges in determining the mechanisms of these compounds in treating atherosclerosis: are the therapeutic effects mediated by an active metabolite alone? Or do several ingredients work together? The questions remain open.

Choosing a suitable research model guarantees the accuracy of the TCM mechanism exploration and the reliability of the results. *In vitro* models, including mouse or human cell lines, are feasible tools to explore cellular functions and mechanisms, as well as gene targets and drug transport. Most of the atherosclerosis-related *in vitro* inflammation models used human or animal-derived macrophage cell lines, such as murine leukemia cell line RAW264.7 and J774 and human leukemia monocyte cell line THP-1. After intervening with phorbol-12-myristate-13- acetate (PMA), 1α, 25-dihydroxyvitamin D3 (vD3), or macrophage colony-stimulating factor (M-CSF), THP-1 could differentiate into macrophages (Chanput et al., 2014). Researchers analyzed the inflammation mechanisms in atherosclerosis by constructing a biology network model and found that HAECs expressed a richer mechanism map compared with immortalized endothelial cell lines (De Leon et al., 2014). Since the macrophages in human and mouse atherosclerotic lesions have been affected by microenvironmental factors, the results obtained from immortal cell lines may differ from the *in vivo* data. Primary macrophages, bone marrow-derived macrophages, and peritoneal macrophages, including large peritoneal macrophages (LPMs) and small peritoneal macrophages (SPMs), are also commonly used cell models in atherosclerosis. Both LPMs and SPMs can coordinate immune responses, but these two peritoneal macrophages subtypes show heterogeneous cellular markers (Lee and Choi, 2020). ApoE^−/−^ mouse model, high-fat diet mouse, and rabbit model constitute the primary *in vivo* platforms for studying underlying pharmaceutical mechanisms in atherosclerosis. However, the location of atherosclerotic lesions in mice is different from that in humans. In the mouse model, plaques locate in the aortic sinus and innominate arteries, while the coronary arteries and carotid arteries are the primary lesions in human (Zhao et al., 2020b; Basu and Bornfeldt, 2020). The experimental models involved in this review can indeed explain the potential mechanisms of TCM in treating atherosclerosis via antioxidants. Nevertheless, due to the limitations of the *in vitro* and *in vivo* models, there is still a long way to go before basic research results can be transformed into the clinic.

In conclusion, translation from the bench to the bedside remains challenging. Oxidative stress is a critical component in the progression of atherosclerosis. Therefore, it is essential to develop medications or supplements to treat atherosclerosis from the perspective of enhancing antioxidant enzyme induction, inhibiting ROS generation, or blocking subsequent reactions, such as inhibition of inflammation process; all of these form vicious cycles in oxidative stress. Further exploration of the therapeutic effect of TCM on atherosclerosis from the perspective of oxidative stress and elucidating the mechanisms and targets will provide reliable evidence for the use of Chinese herbal medicine.
